# Free vascularized iliac bone flap based on deep circumflex iliac vessels graft for the treatment of osteonecrosis of femoral head

**DOI:** 10.1186/s13018-019-1440-2

**Published:** 2019-11-28

**Authors:** Pengfei Lei, Wei Du, Hao Liu, Panfeng Wu, Zhengbing Zhou, Fang Yu, Liming Qing, Ding Pan, Rui Liu, Lei Zeng, Zheming Cao, Qifeng Ou, Juyu Tang

**Affiliations:** 1 Department of Orthopedics, Xiangya Hospital, Central South University, 87 Xiangya Road, Changsha, 410008 Hunan China; 2 Department of Rehabilitation Medicine, Xiangya Hospital, Central South University, 87 Xiangya Road, Changsha, 410008 Hunan China; 3 Department of Surgery, University of Pittsburgh, Pittsburgh, PA 15213 USA

**Keywords:** Osteonecrosis of the femoral head, Young adults, Free vascularized iliac bone flap based on deep circumflex iliac vessels graft, Harris hip score

## Abstract

**Background:**

To investigate the feasibility and clinical efficacy of free vascularized iliac bone flap based on deep iliac circumflex vessels graft for the treatment of osteonecrosis of femoral head (ONFH) in young adults.

**Methods:**

Eighteen patients (19 hips) undergoing ONFH were included from January 2016 to May 2017. After the debridement of the necrotic bones, the contralateral vascularized iliac bone flap was designed and harvested before grafting, in which the deep circumflex iliac vessels and the transverse branch (or ascending branch) of the lateral circumflex femoral artery and their accompanying veins were anastomosed. X-ray was obtained at 1, 3, 6, 9, and 12 months respectively for evaluation of the bone flap healing. Hip function was evaluated with Harris hip score at 18 months postoperatively.

**Results:**

None of the patients is lost to follow-up. All the hips healed well except for four complications: one patient developed superficial wound infection, one patient had subcutaneous hematoma, and two patients developed anterolateral femoral cutaneous nerve injury. X-ray films at 12 months showed improvement in 13 hips (68.4%), five hips (26.3%) were unchanged, and one femoral head collapse with conversion to total hip arthroplasty (THA) at 14 months postoperatively (5.3%). Postoperative mean Harris hip scores were significantly improved compared to the preoperative results (*P* < 0.05).

**Conclusion:**

Free vascularized iliac bone flap based on deep circumflex iliac vessels graft is an acceptable treatment option for young adult ONFH in mid-late stage with low conversion to THA rate at short-term follow-up.

## Background

Osteonecrosis of the femoral head (ONFH), also known as avascular necrosis (AVN), is a refractory and disabling orthopedic disease that typically occurs in young people aged 30–50 [1, 2]. Multiple treatment protocols for ONFH have been followed a comparative analyses of the long-term follow-up results of various treatments for ONFH, confirmed that graft or transfer of the vascularized bone flap (e.g., pedicled iliac bone flap transfer and free vascularized fibular graft) is an effective method for the treatment of femoral head necrosis [3–8].

Pedicled iliac bone flap transfer, with its efficacy being widely accepted, can significantly relieve pain and improve hip function [2]. However, this method has some disadvantages including easily moved vascular pedicle and difficulties in iliac bone flap placement and transfer [9, 10]. Similarly, although free fibular graft is effective in restoring blood supply of the femoral head and providing mechanical support, it is associated with a number of major disadvantages, including trauma to the femoral neck and trochanter, and mismatch between the donor and recipient vessels [11–13]. To overcome these disadvantages, a better operation method to preserve the femoral head remains to be developed.

With the development of small vessel anastomosis, free vascularized iliac bone flap based on deep circumflex iliac vessels graft has also been widely used in the reconstruction of limb and maxillofacial bone defects, due to the better osteogenic ability and speed of bone healing compared to other bone flap graft methods [14].

In this study, we therefore sought a group of patients with ONFH to determine whether free vascularized iliac bone flap based on deep circumflex iliac vessels graft could result in (1) improved Harris hip scores, (2) good bone flap healing, and (3) a low incidence of surgical complications.

## Methods

### Patients

From January 2016 to May 2017, 18 patients (19 hips) who underwent free vascularized iliac bone flap based on deep circumflex iliac vessels graft due to ONFH were included in our research. This study was approved by the Ethics Committee of our Hospital and informed consent forms were obtained from parents. Inclusion criteria were patients who (1) had ONFH with obvious pain, claudication, and other symptoms and failed conservative treatment; (2) were less than 65 years old; (3) had no history of hip infection or hip surgery; and (4) were Association Research Circulation Osseous (Arco) stage II or stage III. A total of 13 men (14 hips) and five women (5 hips) were included, with a mean age of 30.0 years (range 17–49 years). Magnetic resonance imaging (MRI) was routinely used for preoperative evaluation. Preoperative data included X-rays of pelvic area, CT scan, or MRI. According to the Arco classification [15], there were 12 patients in stage II group (12 hips, 63.1%) and six in stage III group (seven hips, 36.8%), respectively. The preoperative Harris hip score of stage II patients was 67.3 points on average, with a minimum score of 58 points and a highest score of 73 points. The preoperative Harris hip score of stage III patients was 58.9 points on average, with a minimum score of 56 points and a highest score of 60 points. The postoperative Harris hip scores were obtained at 18 months postoperatively. For the bilateral hips, the other side of the femoral head started to be treated after one side was completely survived postoperatively. Detailed information on each patient is listed in Table 1.

**Table 1:** Summary of patient data

	Age (yeah)	Gender	Side	Etiology	ARCO stage	Operation time (min)	Blood loss (ml)	Bone flap (cm)	Pre HHS	Post HHS	Follow time (months)	Complication
1	28	Female	L	Glucocorticoid	II	145	170	3*3*2	68	88	33	None
2	48	Female	R	Traumatic	II	150	180	3*3*2	64	79	24	None
3	36	Male	R	Traumatic	II	155	160	4.5*2*3	73	92	19	Infection
4	17	Male	L	Traumatic	II	149	190	4*2*3	58	82	19	None
5	26	Female	L	Idiopathic	II	190	160	4*2*1	72	90	20	None
6	39	Male	L	Traumatic	II	151	180	1*2*2	65	56	25	None
7	26	Male	R	Idiopathic	II	136	150	4*2*2	70	89	20	Hematoma
8	19	Male	L	Traumatic	III	160	150	3*1*1	60	80	22	None
9	40	Male	R	Alcoholic	III	170	230	4.5*2*3	56	78	28	None
10	19	Male	R	Idiopathic	III	205	150	4.5*2*1.5	58	81	20	Cutaneous nerve injury
11	31	Male	BIL	Idiopathic	III/III	172/180	200/190	3*3*2(L)/4*1.5*2(R)	58/60	80/82	19	None
12	17	Male	L	Traumatic	III	183	180	4.5*3*2	60	82	30	None
13	17	Female	L	Glucocorticoid	II	167	230	4*2*2	65	86	22	None
14	46	Female	R	Traumatic	II	170	210	2*3*1	69	88	20	None
15	16	Male	L	Traumatic	II	150	170	2*4*1.5	70	90	18	None
16	49	Male	R	Alcoholic	II	150	150	3.5*2*1.5	64	87	19	None
17	33	Male	R	Alcoholic	III	155	200	4*2*1.5	60	82	26	Cutaneous nerve injury
18	34	Male	R	Idiopathic	II	160	180	3.5*2*1.5	69	87	31	None

### Operative technique

**Fig. 1:**
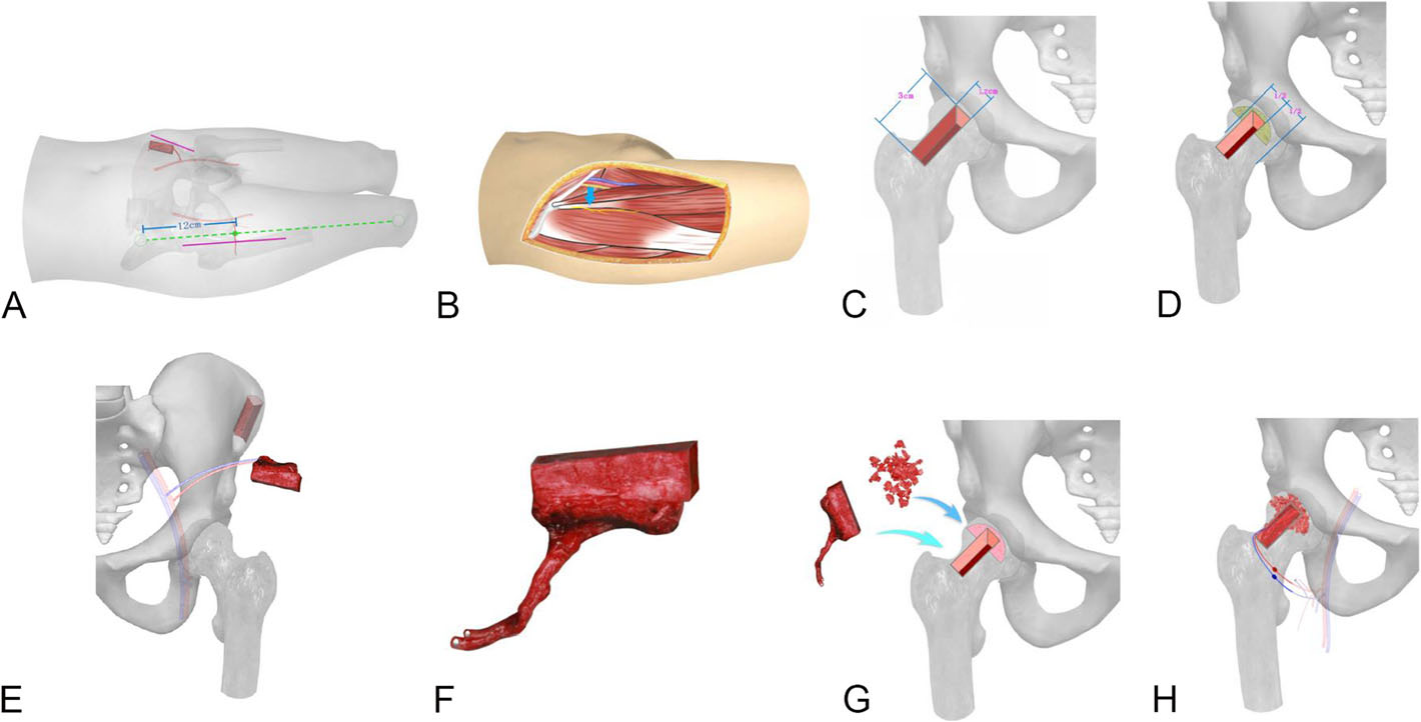
Surgical steps. **a** A 12 cm incision was made along the line between iliac and patella at the starting point of 4 cm below the anterior superior iliac spine of the affected side. **b** The ascending and transverse branches of the lateral circumflex femoral artery emerge were exposed through the intermuscular spaces space between tensor fasciae latae muscle and sartorius muscle and between the vastus lateralis muscle and rectus femoris muscle. **c** The bone window was designed on the femoral neck (about 3 cm × 1.2 cm). **d** Under direct vision, the necrotic lesions were removed with an osteotome and a grinding drill. The articular cartilage surface and the thin layer of subchondral bone were preserved. The femoral neck of the bone window was debrided and the partial capsule was removed. **e** The required length, width, and thickness of the iliac bone flap were measured (typically 4 cm × 2 cm × 1.2 cm). **f** An iliac bone flap of 4 cm × 2 cm × 1.2 cm was harvested, the ramus communicans of the deep circumflex iliac vessels and the iliac crest vessels were cut and ligated. Retrograde dissection was performed until the starting point of the deep circumflex vessel, and the deep circumflex iliac vessels were cut off after confirming that the blood supply of iliac bone was reliable. **g** The iliac bone flap was properly trimmed. After successful trial, the cancellous bone was implanted into the femoral head, and the iliac bone flap was inserted into the bone groove. **h** Under the microscope, the deep circumflex iliac vessels were anastomosed with the transverse branch (or ascending branch) of the lateral circumflex femoral artery and their accompanying veins

The patient was placed in the supine position after tracheal intubation and general anesthesia. All of procedures were performed by one surgery team. MRI and CT scan were performed in bilateral hips. The surgical procedure included removing the necrotic lesions, preparation of the bone window, harvest of the iliac bone flap, graft of the iliac bone flap, and anastomosis of the vessels. A 12 cm incision was made along the line between iliac and patella at the starting point of 4 cm below the anterior superior iliac spine of the affected side (Fig. 1a). The skin and subcutaneous tissue were dissected, and the lateral femoral cutaneous nerve was exposed and protected. The ascending and transverse branches of the lateral circumflex femoral artery emerge were exposed through the intermuscular spaces space between tensor fasciae latae muscle and sartorius muscle and between the vastus lateralis muscle and rectus femoris muscle (Fig. 1b). After partial separation laterally, the distal end was cut and ligated, and the proximal end was clamped with a vascular clamp. Then, the hip joint capsule was dissected in the shape of a cross to expose the femoral neck and the anterior side of the femoral head. The bone window was designed on the femoral neck (about 3 cm × 1.2 cm) (Fig. 1c). Under direct vision, the necrotic lesions were removed with an osteotome and a grinding drill. The articular cartilage surface and the thin layer of subchondral bone were preserved. The femoral neck of the bone window was debrided and the partial capsule was removed (Fig. 1d). The required length, width, and thickness of the iliac bone flap were measured (typically 4 cm × 2 cm × 1.2 cm). According to the measurement data, the iliac bone flap based on deep circumflex iliac vessels was designed and cut out on the contralateral sides (Fig. 1e). The skin, subcutaneous tissue, external oblique aponeurosis, and obliquus internus abdominis were excised and deep circumflex iliac vessels were exposed in the deep obliquus internus abdominis. The deep circumflex iliac vessels were separated and dissected, and the branches along the way were treated with electrocoagulation or ligation to avoid bleeding. The lateral femoral cutaneous nerve, iliohypogastric nerve, and ilioinguinal nerve were then separated and protected. After the deep iliacus was separated, the inner plate of iliac bone as exposed. The iliac bone flap was cut at 2–3 cm lateral to the anterior superior iliac spine, and the outer layer of the iliac crest and iliac bone was preserved. An iliac bone flap of 4 cm × 2 cm × 1.2 cm was harvested; the communication branches of the deep circumflex iliac vessels and the iliac crest vessels were cut and ligated. Retrograde dissection was performed until the starting point of the deep circumflex vessel, and the deep circumflex iliac vessels were cut off after confirming that the blood supply of iliac bone was reliable (Fig. 1f). Part of the cancellous bone of the iliac bone was scraped for use. After complete hemostasis of the donor site wound was conducted, the negative pressure drainage was placed. After the reduction of the iliac crest, the drill hole of the Kirschner wire was sutured with the 2–0 absorbable line. The incision was closed layer by layer and subcutaneous suture was performed. The iliac bone flap was properly trimmed. After successful trial, the cancellous bone was implanted into the femoral head, and the iliac bone flap was inserted into the bone groove (Fig. 1g). Under the microscope, the deep circumflex iliac vessels were anastomosed with the transverse branch (or ascending branch) of the lateral circumflex femoral artery and their accompanying veins (Fig. 1h). After confirming that blood was well ventilated and there was no active bleeding in the wound, drainage was placed and the incision was closed layer by layer.

### Postoperative management

Cefoxitin was used as prophylactic antibiotics during the perioperative period (2 g, ivgtt, twice daily, 48–72 h); papaverine was used to prevent vasospasm 3–7 days postoperatively (30 mg, im, three times a day). The hip-shaped brace was braked for 3 weeks, and the ankle joint active extension, flexion exercise, and the thigh muscle isometric contraction training were performed within 3 weeks postoperatively. Active flexion/extension exercises of the hip and knee joints were started in the bed after removal of brace 3 weeks postoperatively. Postural transfer and non-weight-bearing rehabilitation training were allowed within 4–12 weeks postoperatively. Partial weight-bearing walking with the aid of double crutches was performed 12–24 weeks postoperatively. Twenty-four weeks postoperatively, CT/MRI films were used to evaluate the condition of femoral head healing to decide whether full weight bearing was permitted.

### Clinical assessment and follow-up

The operation time, intraoperative blood loss, and postoperative complications such as delayed incision healing, infection, hematoma, and presence of lateral femoral cutaneous nerve injury were recorded. Hip function evaluation was performed with the Harris hip-scoring system [16]. CT/MRI was performed at all follow up periods to assess revascularization. X-ray films were used to assess bone healing in the iliac bone flap. Femoral heads were assigned to one of three categories postoperatively, based on postoperative X-ray film findings. (1) Improved: necrosis was well healed or was being replaced with new bone, and the joint surface was improved by the effective support of the new bone. (2) Unchanged: compared with the preoperative state, the new bone healed well, the femoral head with cystic or sclerotic changes, or necrotic bone was replaced by new bone, and the joint surface did not show further collapse. (3) Worsening: the joint surface began to collapse, and the necrotic bone was not replaced by new bone.

### Statistical analysis

Statistical analyses were performed using SPSS 19.0 (SPSS Inc., Chicago, IL, USA). Preoperative HHS scores and the final postoperative scores were compared using a paired *t* test. A two-sided *P* value of less than 0.05 was considered statistically significant.

## Results

**Fig. 2:**
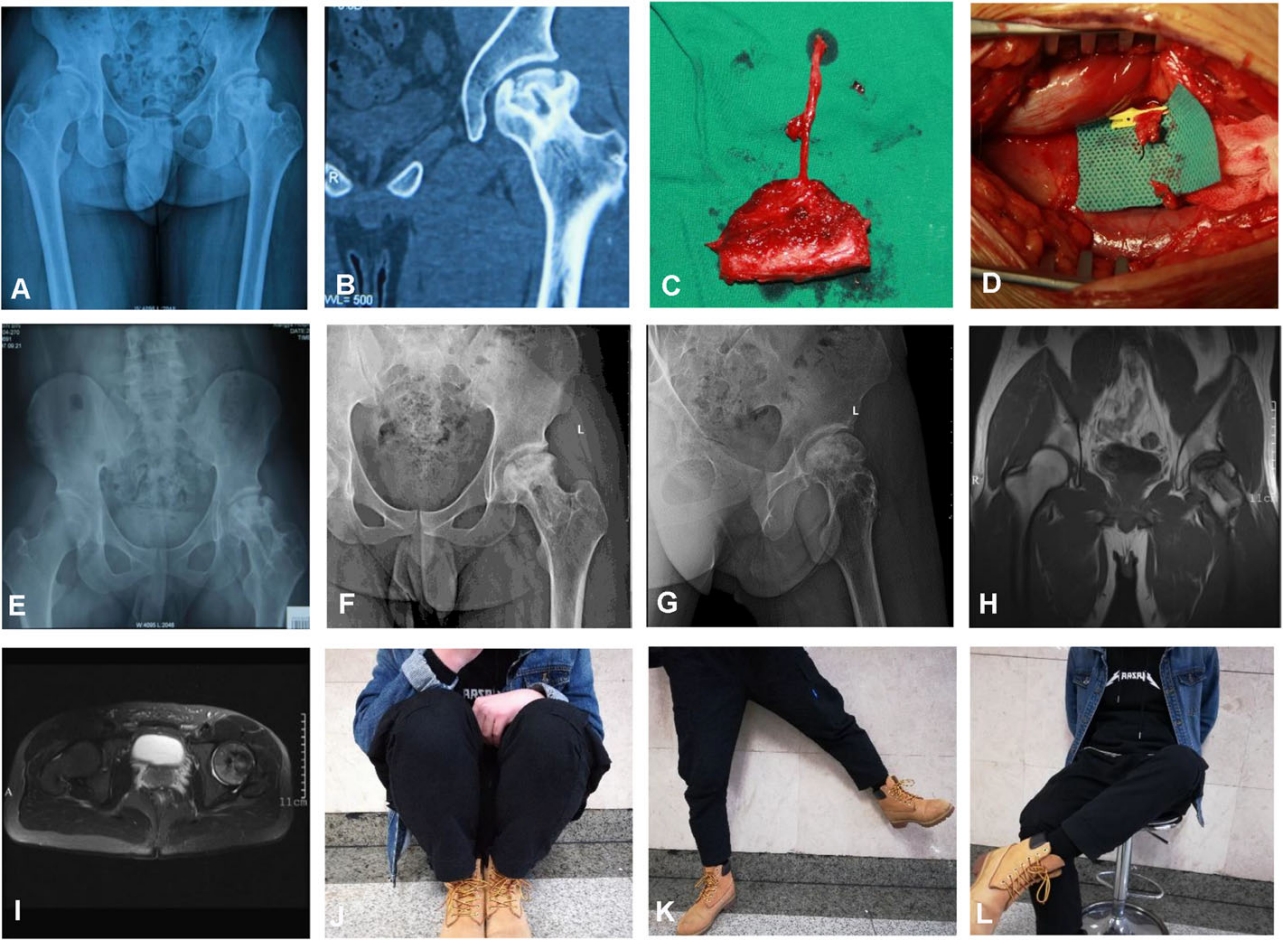
A 19-year-old male patient underwent free graft of vascularized iliac bone flap based on deep circumflex iliac vessels for treatment of traumatic ONFH of the left side. **a** Preoperative X-ray and **b** preoperative CT scans showed heterogeneous density of the femoral head. **c** The iliac bone flap was separated. **d** The cancellous bone was implanted into the femoral head, and the iliac bone flap was inserted into the bone groove. Postoperative anteroposterior (AP) X-rays at **e** 9 months and **f** 27 months, lateral X-rays at **g** 27 months and postoperative MRI scans of **h** AP view and **i** lateral view showed no collapse of femoral heads or narrowing of the hip joint spaces. Hip **j** flexion, **k** abduction, and **l** external rotation showed good recovery

**Fig. 3:**
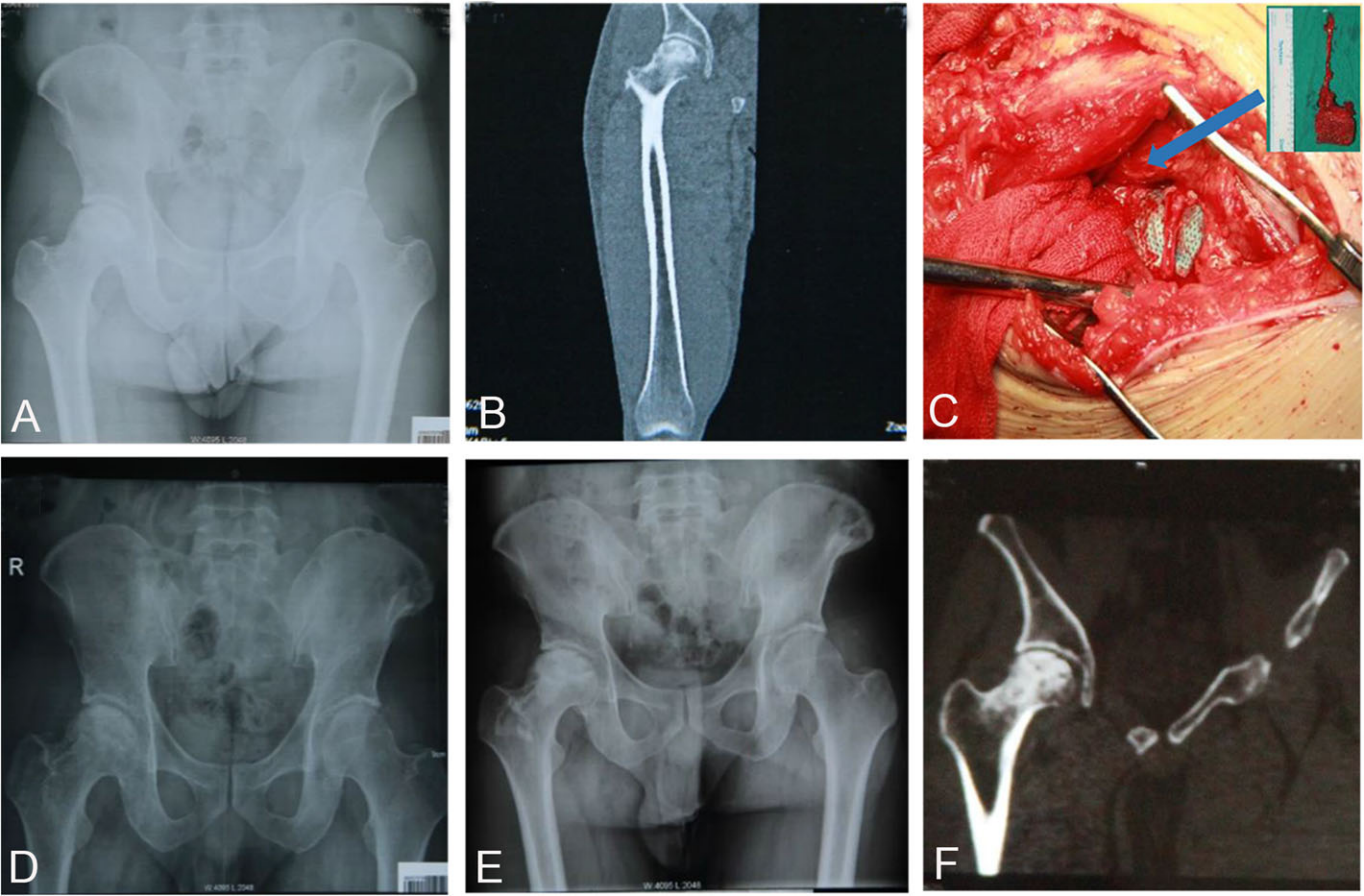
A 33-year-old male patient underwent free graft of vascularized iliac bone flap based on deep circumflex iliac vessels for treatment of idiopathic ONFH of the right side. **a** Preoperative X-ray and **b** preoperative CT scans showed heterogeneous density of the femoral head. **c** The iliac bone flap was separated and implanted into the bone groove. Postoperative X-rays at **d** 3 months, **e** 10 months, and **f** postoperative CT scans at 10 months showed no collapse of femoral heads or narrowing of the hip joint spaces

The mean operation time was 163.1 min (range, 145–205 min). The mean intraoperative bleeding volume was 180.5 ml (range, 150–230 ml). None of the patients were lost to follow-up. All the hips got primary healing except two complications; one developed superficial wound infection and one with subcutaneous hematoma. Two patients (one on the affected side and one on both sides) developed numbness and hypoesthesia of the outer thigh skin without femoral neck fracture and deep venous thrombosis of the lower extremity. After a mean follow-up of 23 months (range, 18–33 months), 18 patients (19 hips) achieved good healing of the iliac bone flap. There was no secondary necrosis and collapse in the remaining 18 hips, except for one hip which developed mild collapse after 2 months of operation. X-ray films at 12 months showed that 13 hips (68.4%) were improved, five hips (26.3%) were unchanged, and one hip (5.3%) was worsening and was converted to total hip arthroplasty 14 months postoperatively. The Harris score was 84.5 for stage II hips and 80.7 for stage III hips, which were significantly higher than those preoperatively (*P* < 0.05). Preoperative and postoperative radiographs of one representative case were shown in Figs. 2 and 3.

## Discussion

Pedicled iliac bone flap transfer is well described since it was first proposed for the treatment of ONFH [17–19]. It can enhance the revascularization of femoral head and the iliac bone provides excellent shape and bulk [20, 21]. However, the pedicled iliac bone flap was difficult to implant under the inguinal ligament and the rectus femoris and the limited vascular pedicle may affect the blood flow of iliac bone flap in some patients [9, 10]. In view of these disadvantages, this article focuses on free vascularized iliac bone flap based on deep circumflex iliac vessels graft for treatment of ONFH.

For free vascularized iliac bone flap based on deep circumflex iliac vessels graft, the transverse branch of the lateral femoral artery was located 10–12 cm below the anterior superior spine and a 10–12 cm incision was started about 4–6 cm below the anterior superior iliac spine. Therefore, the femoral head-neck can be fully exposed and the transverse branch of the lateral femoral artery is approximately in the middle of the incision for easy anastomosis. In addition, the iliac crest and outer plate of ilium were preserved which reduces the damage to the appearance and function. Moreover, the iliac bone flap is embedded into the skin incision. It is stable and reliable without additional internal immobilization and medical expenses. The five cancellous surfaces of the iliac bone flap can fuse well with the femoral head after implantation. As a result, the vascular pedicle is free, the iliac bone flap is easy to inset, and in concert with this, the operation time is diminished and the intraoperative blood loss is reduced compared with the traditional iliac bone flap graft based on deep circumflex iliac vessels.

The most commonly used alternatives to pedicled iliac bone flap transfer are the free vascularized fibular graft. Vascularized fibula can not only strengthen the mechanical property of the femoral head but also benefit the biological microenvironment of the related region [22]. However, for hips in stage III, fibula is difficult to obtain effective filling, and the bone mass and bone quality of fibula flap are worse than the ilium bone flap. With our method, the damage to the femoral trochanter and the femoral neck is reduced, the anastomosis time is shorter, the operation is safer, and the deep iliac vessels are more matched with the recipient vessels compared with free vascularized fibular graft.

The key procedures in our method are the establishment of bone window, preparation of iliac bone flap, and the anastomosis of vessels. The indications and precautions of our surgical method are as follows. (1) Free vascularized iliac bone flap based on deep circumflex iliac vessels graft is suitable for stage II and stage III of ONFH, especially for the treatment of stage III of ONFH. (2) The bone window is prepared above the midline of the femoral neck, which can preserve the integrity of the femoral neck as much as possible and clear the dead bone under direct vision without damaging the calcar femorale. (3) After the iliac bone flap is implanted into the bone window, the separation of the bone flap from the periosteum and the vascular sleeve must be avoided. Therefore, it is necessary to bite off the lateral part of the bone and remove part of the joint capsule, leaving enough space to prevent the vascular pedicle from compression and separation. (4) The size of vascular surface area is only 3 cm^2^ after the distal end, proximal end, and the bottom of iliac bone is 1.5 cm, 0.5 cm, and 0.5 cm embedded in the femoral head respectively. Therefore, the deep circumflex iliac vessels need to enter the iliac bone flap in this 3 cm^2^area to ensure the blood supply of the iliac bone flap. (5) We recommended to harvest the contralateral iliac bone flap so that the muscle cuff can be placed on the lateral superior side and the iliac bone surface can fuse well with the femoral neck surface. Iliac bone flap can be inserted after 180° rotation if the ipsilateral iliac bone flap is essential. (6) One patient in this study restored full weight bearing 2 months postoperatively, resulting in femoral head collapse. Therefore, it is recommended to restore partial weight bearing after 3 months postoperatively. After half a year, a CT should be taken to determine whether full weight bearing is permitted. (7) This procedure has the possibility of injuring the lateral femoral cutaneous nerve in both the receiving area and the donor area. It is required to be clearly exposed and properly protected the nerves during operation to avoid excessive traction injuries. In addition, the iliohypogastric and ilioinguinal nerves may also be damaged when the iliac bone flap is harvested. (8) It is necessary to pay attention to local hemorrhage and prevent the formation of hematoma when the iliac bone flap is harvested; otherwise, it may lead to infection and delayed healing. (9) Considering that the joint space still exists, the main treatment method for collapse cases was to completely remove the necrotic tissue under direct vision and design the iliac bone flap based on deep circumflex iliac vessels for precise repair. The advantages including strong osteogenesis and fast healing of this method could keep the femoral head from collapsing again.

One of the limitations of our method is the complications which may be attributed to the surgery trauma and blood loss caused by the cutting of iliac bone flap. In our study, two cases of lateral femoral cutaneous nerve injury, one case of hematoma, and one case of infection occurred, which delayed the overall recovery of those patients. Another limitation is the short follow-up periods and small sample size; therefore, large randomized controlled study should be carried out in the future to further explore long-term efficacy.

## Conclusion

Free vascularized iliac bone flap based on deep circumflex iliac vessels graft is a promising treatment for ONFH in mid-late stage. Further supporting studies are needed to confirm the success achieved in this study.

## Abbreviations

AVN: Avascular necrosis
MRI: Magnetic resonance imaging
ONFH: Treatment of osteonecrosis of femoral head
THA: Total hip arthroplasty
